# Analyzing efforts to synergize the global health agenda of universal health coverage, health security and health promotion: a case-study from Ethiopia

**DOI:** 10.1186/s12992-021-00702-7

**Published:** 2021-04-26

**Authors:** Amare Worku Tadesse, Kassu Ketema Gurmu, Selamawit Tesfaye Kebede, Mahlet Kifle Habtemariam

**Affiliations:** 1 Department of Infectious Disease Epidemiology, London School of Hygiene and Tropical Medicine, London, UK; 2 Department of Reproductive Health, Nutrition and Population, Addis Continental Institute of Public Health, Addis Ababa, Ethiopia; 3 Department of Global Health and Policy, Addis Continental Institute of Public Health, Addis Ababa, Ethiopia; 4 Africa Centers for Disease Control and Prevention, Addis Ababa, Ethiopia

**Keywords:** Synergies, Fragmentation, Health systems, Ethiopia

## Abstract

**Background:**

Evidence exists about synergies among universal health coverage, health security and health promotion. Uniting these three global agendas has brought success to the country’s health sector. This study aimed to document the efforts Ethiopia has made to apply nationally synergistic approaches uniting these three global health agendas. Our study is part of the Lancet Commission on synergies between these global agendas.

**Methods:**

We employed a case study design to describe the synergistic process in the Ethiopian health system based on a review of national strategies and policy documents, and key informant interviews with current and former policymakers, and academics. We analyzed the “hardware” (using the World Health Organization’s building blocks) and the “software” (ideas, interests, and power relations) of the Ethiopian health system according to the aforementioned three global agendas.

**Results:**

Fragmentation of health system primarily manifested as inequities in access to health services, low health workforce and limited capacity to implementation guidelines. Donor driven vertical programs, multiple modalities of health financing, and inadequate multisectoral collaborations were also found to be key features of fragmentation. Several approaches were found to be instrumental in fostering synergies within the global health agenda. These included strong political and technical leadership within the government, transparent coordination, and engagement of stakeholders in the process of priority setting and annual resource mapping. Furthermore, harmonization and alignment of the national strategic plan with international commitments, joint financial arrangements with stakeholders and standing partnership platforms facilitated efforts for synergy.

**Conclusions:**

Ethiopia has implemented multiple approaches to overcome fragmentation. Such synergistic efforts of the primary global health agendas have made significant contributions to the improvement of the country’s health indicators and may promote sustained functionality of the health system.

## Background

Ethiopia, a low-income country in Eastern Africa, ranks 173rd on the United Nations Development Program (UNDP) Human Development Index [1]. With a projected population of 109 million in 2020 and growth rate of 2.6%, half of the population is under 15 years old and the average life expectancy at birth has increased to 66.2 years [1].

Ethiopia has decentralized health care governance and delivery structured into a three-tier system. This enables the government to examine the effectiveness, efficiency, equity and sustainability of health services as well as foster engagement of local stakeholders through policy dialogue [2]. Ethiopia’s health sector has been guided by a national health policy, translated into action through a 20-year long series of five-year plans known as the Health Sector Development Plan (HSDP) (1995–2015) and the Health Sector Transformation Plan (HSTP) post 2015. Ethiopia aspires to achieve universal health coverage by 2035 [2].

Ethiopia’s health strategies primarily focus on creating access to primary health care services, improving equity and quality of health care through utilization of essential health services, and building community ownership. These strategies have resulted in improving health outcomes of the population, as evidenced by achieving most of the health-related Millennium Development Goals (MDGs), and remarkable progress in the other non-health MDG indicators [2]. Building on these successes and on a global focus on sustainable development, Ethiopia aligned its HSTP impact targets with the Sustainable Development Goals (SDG) to meet the SDG targets by 2030 [2].

Over the past two decades, the government has implemented comprehensive social and economic reforms. A key feature of this reform has been the government’s strong commitment to shifting its spending to sectors such as health, education, road infrastructure, agriculture, and rural development that, if strengthened, will reduce poverty [3]. The presence of a strong health system is critical to ensuring universal access to quality and affordable health care, protect people from health emergencies, and encourage them live healthier lives [4, 5]. However, efforts addressing these three areas are fragmented. Fragmentation across health system structures, healthcare funding, and global health actors’ engagement has been widely reported in global health literature [6–9]. This fragmentation has resulted in suboptimal care, higher cost due to duplication of efforts and resources, reduced efficiency, and poor quality of care, and it has negatively affected efforts to strengthen health systems. Multiple reasons were provided for this fragmentation, including a tendency among global actors towards vertical programs [6, 9, 10]. Such fragmentation is particularly common in low- and middle-income countries, and Ethiopia is no exception.

In contrast, there is evidence supporting the existence of synergies among universal health coverage, health security and health promotion [4, 11] and these three priorities are mutually reinforcing, not mutually exclusive [12]. Health system strengthening comprises the means (the policy instruments), while universal health coverage is a way of framing the objectives of policy. This policy identifies that individual health security provides the intrinsic value of protection against risk, including disease prevention and health promotion [4, 5]. Collective health security, reducing the vulnerability of societies to health threats that spread across national borders, is a goal that extends beyond the definition of universal health coverage. Health security traditionally emphasizes the role of health system capacities and technical areas in the prevention, detection, and response to emerging and re-emerging infections. Health system strengthening, on the other hand, is often conceptualized in the six building blocks of the WHO health system framework [5].

To date, no study has comprehensively documented Ethiopia’s efforts at utilizing a synergistic approach toward the three main global health agendas. It is believed that documenting this experience may help other low- and middle-income countries learn from Ethiopia. Our questions and outcomes were driven by assumptions of the Lancet Commission on synergies between universal health coverage, health security and health promotion to improve health and equity for all people worldwide [13]. Therefore, this study specifically aimed to document Ethiopia’s efforts to align universal health coverage (UHC), health security (HS) & health promotion (HP) and their implementation within its health system. Furthermore, we aimed to understand Ethiopia’s efforts to create synergies through health systems analysis, and to identify social, political, and economic conditions that may have facilitated synergy in Ethiopia.

## Methods

Case study is a comprehensive method that incorporates multiple sources of data to provide detailed accounts of complex research phenomena in real-life contexts [14]. This study employed a case study design as it was found to be of significant advantage to explore the answers to “what”, “why” and “how” questions. In addition, the study described a real-life context where the process has already happened, thus, the researchers have no control over the events.. This study described how decisions were made in the health sector. Multiple data sources, including strategy and policy documents, and key informant interviews, were used to support data triangulation [15] and ensure the rigour of the study. Only reports and information written in English language were considered. The Government of Ethiopia’s policy and strategy documents and reports were included in the review. In addition to documentary evidence, purposive and snowballing techniques were employed to select key informants for interviews, including previous and current policymakers, and academics in the Ethiopian health system. We initially planned to include nine key informants to participate in the study, however, saturation was reached after six respondents. Respondents were women (*n* = 2) and men, and held senior managerial, technical, and academic research positions. All study respondents had extensive experience on the topic we examined and a profound knowledge on the issues of fragmentation at the national and global level, which allowed us to efficiently capture key areas relating to our study aims. The authors collaboratively analysed the document reviews and interviews. Following initial familiarization with the data, the WHO building block framework was identified as most appropriate to analyze the data by including relevant respondents’ quotations. A framework analysis containing both the “hardware” and the “software” of the health systems, which has been previously used in health policy and systems research [15, 16], were employed to analyse the Ethiopian health system for synergistic approaches to UHC, HS and HP.. The WHO Health Systems Framework was developed to provide a model to capture the interlinked and complex nature of health systems [4]. This framework was used to guide the development of data collection tools and data analysis. The “hardware” of the health system includes the six core components or WHO’s building blocks of the health system: (i) service delivery, (ii) health workforce, (iii) health information systems, (iv) access to essential medicines, (v) financing, and (vi) leadership/governance. The “software” of the health system includes “the ideas and interests, values and norms, and affinities and power that guide actions and underpin the relationships among system actors and elements” [16–18].

The study protocol was approved by the Addis Continental Institutional Review Board (Ref. No. ACIPH/IRB/005/2019).

## Results

### COVID-19 and the Ethiopian health system

The pandemic of Coronavirus Disease 2019 (COVID-19) is a timely reminder of the nature and impact of emerging infectious diseases that are public health emergencies (PHEs) of international concern. Various approaches, strategies and programs have been developed to address PHEs at national and global levels, including initiatives to strengthen public health preparedness and global health security (GHS). These represent the proactive and reactive efforts required to protect the world’s population from PHEs [19]. Ethiopia is a resource-limited country in Sub-Saharan Africa with a challenged health system, limited Intensive Care Unit (ICU) capacity, and a fragile economy [20].

Since the COVID-19 pandemic was reported, Ethiopia started screening air travelers, initially sending laboratory specimens abroad for confirmation of suspected cases. With the support of the WHO, the Ethiopian Public Health Institute (EPHI) started laboratory test for COVID-19 on February 7, 2020 [21]. Along with capacitating the health system, the country initially dedicated certain hospitals for treatment of COVID-19 cases. With further progress, treatment and testing facilities were (and continue to be) expanded to cities outside the main capital [21].

Ethiopia reported the first COVID-19 case on March 13, 2020 [22], followed by early imposition of stringent measures on March 23, and a declaration of a 5 month state of emergency on April 10th. The country opted for a balanced approach, where industry and agriculture have continued to operate, and steps to mitigate the effects of the measures on the most vulnerable were undertaken by both the government and non-governmental organizations [20, 23]. However, the impact of COVID-19 could continue to cause greater loss of life in Ethiopia if efforts are not directed at mitigating interruptions of essential healthcare services and clearly addressing behavioral and sociocultural norms that would facilitate the spread of the disease [24, 25].

### Fragmentation in the Ethiopian health system

Government ministries, bilateral organizations, United Nation (UN) agencies, non-governmental organizations, civil service organizations and training institutions all play an important role in the Ethiopian health system [2]. Government organizations, UN agencies, donors and the community have put UHC and HP at the forefront of their policy priorities. The Global Fund to Fight AIDS, Tuberculosis and Malaria (GFATM), United States Center for Disease Control (US CDC) and the Ethiopian Federal HIV/ AIDS Prevention and Control Office (FHAPCO) have strong positions within the health security agenda and high levels of influence.

Universal health coverage was perceived as an equity umbrella term that was all about leaving no one behind, from health promotion to palliative care. Health security was also seen as part of universal health coverage, and both need a strong health system. This underscored the importance of aligning efforts directed at the three global agendas within national health policies, plans and their implementation. Fragmentation in the Ethiopian health system had posed critical challenges to the nation’s efforts on improving key health indicators. This has been evidenced by inadequate multi-sectoral collaboration, poor integration of surveillance functions and service delivery, and inadequate resource mapping and implementation capacity. Other reasons for health sector fragmentation in Ethiopia include global actors’ tendency towards vertical programs, poor public private partnership, resource constraints, lack of multi sectoral collaboration, lack of professional motivation, inadequate capacity of the government to plan/set direction, and gaps in leadership skills. As a result, efforts addressing the three global agendas – universal health coverage, health promotion and health security – had been fragmented.

*“the universal health coverage and health promotion goes hand in hand, but the health security goes separate as it was linked to responses to health emergencies only …” KII_4*

#### Governance and leadership

Multiple donors had established their own parallel planning, implementation modality, and monitoring, accounting, and reporting mechanisms. At times, the annual budget closing period of the donors differed from the Ethiopian Ministry of Health’s financial procedures, which resulted in duplication of effort and additional workload for health care providers and staff at the ministry of health [26].

*“the ministry of health had more than hundred accounts because they all had different financing schemes” KII_2*

*“… the national policy documents clearly reflect the three global agendas* (UHC, HS, HP) *but non-compliance to guidelines have been reported during implementation efforts” KII_6*

Furthermore, there had been intersectoral fragmentation between Ministries of Health, Education, Agriculture, Water, Electricity and Transport which was evident during national initiatives, including health facility expansion, curriculum revision, and food security programs aimed at bringing universal health coverage and health promotion to the community. These had resulted in poor facility and infrastructural setup, poor quality pre-service training, high rate of unemployment among health workforce and high rate of stunting which could largely be attributed to poor multi-sectoral collaboration.

*“… In Ethiopia, human capital index has been calculated and it shows an individual’s potential ability to work, based on age, is only 38% which can be attributed to multi sectoral fragmentation” KII_3*

Ethiopian overall UHC service coverage for the year 2015 was about 34.3%. This coverage was considerably lower compared with the global average service coverage (64%) and sub-Saharan Africa average (42%) [27]. In Ethiopia, though health promotion has been part of every program, health security was not included as part of the agenda. One example is that actions undertaken during a health emergency were unorganized and fragmented until the establishment of Public Health Emergency Management (PHEM) under Ethiopian public health institution (EPHI) in 2009.

*“Many organizations, including WHO, have viewed health security as separate structure … the advantage is it helped to give immediate response, but it (health security) has to stay (integrated to) in the system by creating operational procedure...” KII_2*

#### Health service delivery

Another form of fragmentation was seen among disease control programs. For example, the national immunization program followed the protocols of GAVI (Global Alliance for Vaccines and Immunization) when it came to planning, implementation modalities, accounting mechanisms and reporting. Similarly, programs supported by the World Bank and Global Fund, such as malaria and tuberculosis (TB) programs, each utilized their own protocols.

*“In my opinion, there are still some programs that continue to operate discretely without reflecting integration efforts of the national health system” KII_6*

In addition, service delivery activities at the health facility level were fragmented. Maternal health, HIV/AIDS, TB and malaria tended to be more vertically oriented, and related activities were not integrated. When a TB patient who additionally has a skin disease like scabies goes to the TB clinic, the health provider does not give much attention to treating the scabies due to the verticality of the system, resulting in a negative impact on beneficiaries. Furthermore, poor integration of services lowers the quality of health care services which could eventually lead to communities’ poor acceptance of health care provided by the government.

*“majority of our programs are not like one stop shopping; one may be directed to another department or may be told to return back another day to get the service…” KII_5*

#### Health workforce

Most in-service trainings were provided by development partners. They were using their own training materials and methods, and utilized different durations of training. The trainings were not need-based or pre-planned, and there were no quality assurance mechanisms, monitoring and evaluation plans, or planned follow-ups after training. There was no national database indicating the number and mix of health professionals disaggregated by type and level of training.

*“Due to poor planning for pre-service trainings, at some point, we encountered shortage of pharmacists and laboratory technicians in the market” KII_1*

#### Health system financing

The per capita health expenditure, at 31 USD [28], makes Ethiopia one of the several countries in Africa which falls short, far below the international health expenditure benchmarks and targets [29–32]. In addition to the insufficient overall funding for health, the government’s contribution is still far from the Abuja commitment of 15% [33]. In 2018, the share of health expenditure from the total public government expenditure amounted to about 8.2%. According to the sixth national health accounts [34], 36% of health sector financing was supplied by foreign assistance, 30% by government and 33% were out-of-pocket expenses borne by the patients. On average, the government manages 45% of the total funding for health resources in a given time. A closer look at disease-specific out-of-pocket (OOP) expenditure revealed that the financial burden from the different sources varied significantly across disease entities. sOut of pocket spending illustrates one of the most severe forms of fragmentation, as it places the burden of health care funding on an individual, resulting in health service use distribution according to ability to pay instead of need. The OOP was the lowest for reproductive health (15%), followed by infectious diseases (31%), followed by non-communicable diseases (NCDs) (70%) and nutritional deficiencies (77%). On the other hand, contribution to NCDs from development assistance was negligible at 2% whereas 50% of financing for infectious diseases is covered by aid. On average, 42% of total health spending goes to primary and preventive care [34]. Even though the community-based health insurance aims address equity and contribute to UHC, the fund was not pooled at the regional level, which resulted in inadequate funds and led to higher community out-of-pocket (OOP) expenses.

“*…if it (the money) was pooled at regional level, the amount would have increased but now when they (the sick) go to health facility, facilities may not have drugs (medicine). So, the community is forced to use out of pocket payment … it was scaled up without modification after impact assessment …” KII_3*

**Fig. 1:**
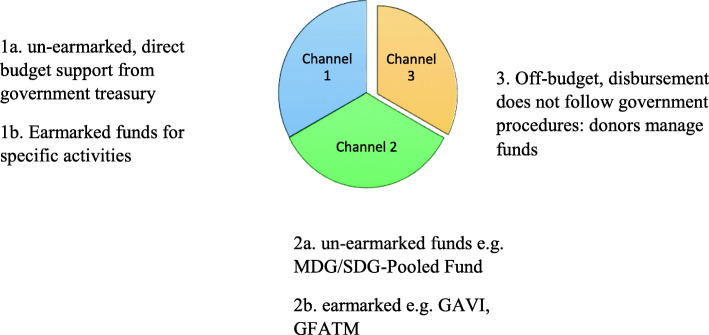
Funding channels for donor finance in Ethiopian health system

Significant funding for the Ethiopian health sector comes from foreign assistance via three different channels: channel 1, channel 2 and channel 3 (Fig. 1). Budget allocation was influenced by donors’ interest and a significant portion went to channel three which flows to Non-Governmental Organizations (NGOs) and Civil Society Organizations (CSOs). A significant portion of donor financing is directed through Channel 3, off-budget, which also goes to the prevention and control of communicable diseases. The other significant portion comes through channel 2b earmarked for vaccines, HIV/AIDS, TB and Malaria interventions.

#### Access to essential medicines

The Ethiopian government procures necessary medical supplies through Ethiopian Pharmaceuticals Supply Agency (EPSA), the former Pharmaceuticals Fund and Supply Agency (PFSA). There were two schemes for procurement of commodities that were necessary for providing a health service: program commodities like medications to treat TB and malaria, and non-program commodities like anti-pain medications. Donors monitor and support program commodities closely but there was no monitoring system for non-program commodities and stock outs was more frequent for non-program commodities than program commodities. Sometimes the procurement system does not go in alignment with the regulatory function. One respondent reported that some supplies had been imported by donors without assessing the demand which resulted in wastage due to short shelf life and imbalanced medical drug distribution. This, in turn, forced clients to pay out of pocket for medical drugs. Weak management practices played a major role in fragmenting equitable access to the health system due to lack of a clear plan and set of goals from the government.

#### Health information system (HIS)

Resources for developing a HIS had been channeled through various implementing partners, which resulted in multiple, fragmented approaches [35]. The routine monitoring activity indicators and reporting mechanism was bulky, fragmented, non-standardized and non-systematic. There were different reporting formats which allowed parallel reporting until the Ethiopian ministry of health took the leadership and harmonized the reporting schemes. Establishing Health Management Information System (HMIS) at all levels of health service delivery system and setting up HMIS units at all levels for ensuring information use for evidence-based health planning and decision-making were the major targets that were set during HSDP-II [36]. In the fragmented system, the government could not set priorities and ensure equity, leading to unmet social needs and poor quality of health care service which, in turn, affected health outcomes. Inefficient utilization of the available limited resource to set priority agendas led to resource wastage and duplication of effort.

*“fragmentation of the system competes with patient care time as health care providers have to fill lots of forms, which in turn decrease quality of care the doctors provide to the patient.” KII__2*

### Efforts towards synergy in the Ethiopian health system

The Government of Ethiopia is committed to improving the national health system. Ethiopia, as an early signatory of the International Health Partnership plus (IHP+) compact, endorsed the principles of harmonization and alignment which are in line with the Paris declaration for aid effectiveness. Thus, Ethiopia has made marked improvements in many health indicators, mostly through a well-coordinated effort and intensive investment in primary health care by the government, development partners and the community at large [2].

*“the focus on primary health care was one of the facilitators for the defragmentation* (synergy) *of the Ethiopian health system” KII_3*

Even if there were previous efforts at synergy, the first effort at facilitating synergies in the health system started in 2011 along with the roadmap that was developed to improve harmonization and alignment in the health sector by “enhancing one plan, one budget and one report”. This roadmap identified the main activities to be implemented, timeline and modalities of implementation, responsibilities of the government, development partners, implementing partners and other stakeholders, and the monitoring and evaluation of the road map [26]. Furthermore, the development of the Information Revolution Roadmap (IRR) led by FMOH to support the governing bodies coordinated the efforts of donors and implementing partners working on health management information systems in Ethiopia to ensure they are supporting one unified system [37].

#### Governance and leadership

The cornerstone of the synergistic efforts of the Ethiopian health system was strong political will and commitment, led by those at a high level in the Ministry of Health and even beyond. It was ultimately led by the office of the prime minister, who gave the highest attention to health.

*“For defragmentation* (synergy)*, there must be a body responsible for setting agenda at a country level, which has to be country owned and led*…. *Government should also play its leadership role to meet the global commitment to present health as a right for the community” KII_5*

While the Federal Ministry of Health was responsible for the formulation and harmonization of health programmes and strategies, the Regional Health Bureaus (RHBs) were mostly responsible for actual implementation [27]. Based on the key informants’ reports, several donors’ assessments of the health system around 2005 revealed fragmentation of the Ethiopian health system mainly due to weak leadership and uncoordinated actions. The Ethiopian Ministry of Health took the lead in implementing synergies in the health system. It started with restructuring the multiple budgeting and reporting schemes to one-plan, one-budget and one-report at all levels of the health system. They also closed several bank accounts used by the ministry to streamline the finances and increase effectiveness, which played an important role in improving harmonization and alignment in the Ethiopian health sector.

*“previously every donor had its own plan but now every partner makes plans based on ministry of health plan” KII_4*

The Ministry of Health established a platform to engage donors from planning to policy formulation through the Joint Consultative Forum (JCF) led by the Minster of Health and Joint Core Coordinating Committee (JCCC), led by the Ministry’s plan and policy department. Through this platform, the partners’ engagement spanned from resource mapping at a national to district level with resulting detailed financial report [38]. Furthermore, a joint review committee monitored specific activities/programmatic challenges, lesson learned on implementation, and governance and finance schemes from a national to sub-district level to evaluate and validate the effectiveness of the programs. Development of strategic documents to implement the health policy were rolled out in a series of five-year plans with a clear set of goals aligned with primary health care. The joint assessment for national strategy (JANS) committee involved the WHO and other partners/ stakeholders including professionals to assess the developed plan and to improve the quality, thus fostering improved synergies in the health sector.

*“partners work in collaboration, they know the plan, target, and have agreed on where and on what they work on every year” KII_3*

Furthermore, continuous system reforms, including institutionalization of Business Processing and Reengineering (BPR), and district-based planning played important roles in the synergizing efforts.

#### Health service delivery

In 2002, Ethiopia introduced the Health Extension Program (HEP) at the community level to promote UHC. Such community-based care has improved access by moving primary care services to the community level to reach more people by making their homes and villages the point of care. The HEP has been serving as a primary vehicle for prevention, health promotion, behavioral change communication, and basic curative care through effective implementation of the 16 health packages. These packages were framed under four main themes: (i) health education and communication, (ii) hygiene and environmental sanitation, (iii) disease prevention and control and (iv) family health [36].

The Essential Package of Health Services (EHSP), introduced in 2005, was aligned and built on the existing HEP. EHSP was developed with an intent to have public sector facilities provide a minimum standard of care that fosters an integrated service delivery approach essential for promoting the health of the population [39]. Priority reproductive, maternal, newborn and child health (RMNCH) interventions, such as family planning, antenatal care, postnatal care, immunization, integrated management of childhood neonatal infections, essential nutrition actions, and treatment of communicable diseases, are provided at the three-tier health system. These services were delivered through government-sponsored community health workers, and public sector primary care and referral facilities targeting women, adolescents, the indigent and rural populations to improve equity [39]. The integration saved not only money but also time for procurement and the one reporting system decreased duplication of individual effort, which translated into a focus on health care service delivery.

*“Health promotion has become a component of the health extension program and strengthened community-based health services” KII_1*

Initially, Ethiopia had limited capacity to handle emergencies and health security was not prioritized. Eventually, it became an individual agenda item and ultimately was organized under Ethiopian Public Health Institute (EPHI) as Public Health Emergency Management (PHEM). Previously, there was little organization around emergencies and strategies were fragmented. Since formation of PHEM, with its own human resources, guidelines and protocols, we now have a better and more cohesive way to address emergencies and improve health security.

*“As you can see PHEM is giving the necessary information regarding the corona virus….it is timely, well organized and structured” KII_1*

#### Health workforce

The presence of sufficient, fairly-distributed, competent, responsive and productive human resource is a core requirement to ensure the agenda of universal health coverage, health promotion, and health security.

*“Previously the ministry of health followed a specialist approach… if you were trained on malaria, you only work on malaria but now, it (MoH) follows a generalist approach” KII_4*

To address the fragmented recruitment, deployment, and distribution of health workforce, Ethiopia has taken several steps. It has developed a national strategy, which resulted in the re-structuring of the human resource department. One of the directorates that resulted from this restructuring was the human resource development directorate that leads the planning, training, recruitment, deployment, and development process that ensures that all health professionals are recruited and deployed using standardized criteria. There is an in-service training unit that has developed national in-service training guidelines and a standardized checklist that is to be used by all parties who are involved in development of training packages. This unit routinely reviewed and approved all national training packages for technical content, training approaches and methodology, duration, and quality assurance mechanisms, ensuring that all in-service trainings are standardized.

#### Health system financing

An area which benefitted from significant synergy was health care financing. The MDG pool fund exemplified these efforts the context of the global health agenda. it enabled the government to allocate funds for a prioritized national agenda. When the resources were pooled, allocation is based on the prioritized agenda which enabled Ethiopia to address health equity and quality. Improving the service quality led to healthier and more productive communities that increased community satisfaction and better supported the country’s economy.

In addition, to build capacity and leadership within the ministry, many organizations provided seconded staff and the activities of the ministry became result oriented. The ministry of health encourages a corruption-free health system by promoting transparent coordination, establishment of strong technical working groups and grant management committees that builds donors’ trust.

*“All the resources were put in one account for management, which created the MDG pool fund…... those organizations who couldn’t use the pool fund due to their nature came to one plan and indicated what they want to work on” KII_2*

Although health insurance was introduced in 2010, the policy has a longer history. The government noted insurance as a possible source of finance in the 1993 Health Policy and 1998 Healthcare Financing Strategy (HCFS) [40]. Community-based health insurance and service fee waivers for specific care, such as TB, pregnancy, family planning, HIV/AIDS, leprosy, fistula, and epidemics, are mechanisms to ensure health services reach to the target population [41]. Community-based health insurance is an investment and, at the same time, a process of optimization to provide equitable quality service. It aims to address universal health coverage whereby the government contributes for indigents so they can utilize health services free of charge through a pooled national fund. However, the pooled fund is at a district level and discussions are currently happening to expand the pool to a regional level. In addition, the ministry highly considered innovative way of financing health care, such as an excise tax levied on certain goods including alcohol, tobacco, and sweet beverages, and how much of the collected tax should be allocated for health to prevent the community from high out-of-pocket expenditures.

*“Insurance is all about spreading financial risk…, the greater the pool, the more people can be protected.” KII_5*

#### Access to essential medicines

Efforts were also made to provide a well-functioning medical supply system to ensure equitable access to essential, safe and quality medical products, vaccines and technologies. This has been mainly apparent in efforts to improve proximity and efficiency in the distribution of supplies to ensure universal health coverage**.** To safeguard this, efforts were undertaken to integrate the pharmaceutical supply system to the health care system through the integrated pharmaceutical logistic system (IPLS).

*“strong forecasting and quantification exercises are vital to address the stockout at facility level” KII_5*

#### Health information system

The Information Revolution Roadmap (IRR) allowed for a clear governance framework to be put in place to align donor agencies, implementing partners, and the various directorates and agencies of the FMOH, leading to unanimous stakeholder support of a single HIS based on District Health Information System 2 (DHIS2) [35]. Strong leadership within the government with global acceptability encouraged donors to align their health information system reporting mechanisms and standardization and interpretation of indicators. Furthermore, participation of development partners and programs in revising, and regularly reviewing and providing feedback in the process of the health management information system has significantly reduced parallel reporting [2].

*“effort was made to reconcile our reporting mechanism, set of indicators and calendar…...now we have a relatively better routine reporting mechanism and system”. KII_1*

The ministry of health worked closely with regional health bureaus and district health offices on the entire health information system, from planning to reporting. The ministry endorsed a top-down and bottom-up approach to plan and develop a set of health indicators which were sent to regional health bureaus for further enrichment and then to districts for development of an actual detailed plan. The ministry prioritized and selected 10-12 indicators for close follow-up and discussion every month and met quarterly with the regional health bureaus. This approach facilitated the ministry’s effort to address the geographical accessibility of community-based health interventions, including the health workforce development, in terms of numbers and types of professionals, and promoted the sectoral coordination and collaboration mechanism.

*“It (essential health package) is used as a communication tool so partners even the government when allocating resources will use the document as a reference…...Defining essential health packages contributes a lot for an integrated approach” KII_5*

### The “software” of the health system

Powerful interests supported creating synergies in Ethiopia. One potential interest was the former minister of health and foreign affairs of Ethiopia/current director general of the World Health Organizations, and his successor at the Ministry of Health. Both have been supporters of synergy efforts. Other powerful interests which were supportive of synergy include the Universal Health Coverage Partnership (which was previously called International Health Partnership (IHP+). Ethiopia showed efforts to improve community ownership and trust through health extension programs by empowering the community with health information, community conversations, and building strong patent-provider relationships, and through activities like awarding model families, *kebeles –* the smallest administrative unit in Ethiopia – and *woredas* (districts).

*“if facilities deliver what they planned, government gains trustworthiness and communities show increased health seeking behavior” KII_2*

The Health Sector Transformation Plan, and its predecessor, the Health Sector Development Plan, identified harmonization and alignment as the core values and guiding principles of the health sector. The government showed its strong commitment to harmonization and alignment by developing and signing the Harmonization Manual in 2007. This manual employs a three-tier collaborative governance system made up of the Central Joint Steering Committee, the Joint Consultative Committee, and the Joint Core Coordinating Committee. It also lays out a provision for an annual review meeting where annual plans and performances are endorsed, a vision of one-plan, one-budget and one-report, a three-channel funding mechanism, and agreed upon indicators [38]. These values and principles have played a significant role in the creation of synergies between the three global health agenda [2, 18].

### Challenges that affected synergy efforts

Despite huge efforts in forging synergies between the main global health agendas in the Ethiopian health system, it has been challenged by several barriers. These barriers include frequent turnover of health workforce, resulting in repeated training sessions and donor dependent financing leading to inequity, regional administrations’ autonomy, and poor coordination around previously identified priorities. Even though the government is increasing the budget for health, donor dependency is still very high, affecting sustainability. It was difficult to bring equity, universal health coverage and quality without the government gaining a financial lead. Generally, financing for health was characterized by a substantial funding gap evidenced by the HSDP IV and HSTP I having close to 50 and 30% funding deficit respectively [36].

Other barriers to synergy efforts in the Ethiopian health system include lack of professional motivation, poor facility infrastructure, and less emphasis given to private and civil society organizations. There is also a gap in the procurement and supply chain management of medical supplies at the facility level, and in forecasting stockouts [41]. While partners focus on providing technical assistance on forecasting their program commodities, less emphasis has been placed on non-program commodities. In addition, variable health workforce in terms of number, skill mix, competency and motivation, was challenging the health system.

*“The main problem across all regions include inadequate human resource, high attrition and demotivated staff … unless the government solve this problem, it is a very difficult challenge in the health system …*” KII_3

*“the information that comes from the HMIS is bulky and achievements often seem exaugurated? … as a result, the donors conduct multiple surveys to confirm the results which leads to wasting the available limited resources.” KII_3*

## Discussion

### Summary of main findings

The major forms of fragmentations in Ethiopian health system had been identified as planning, budgeting, human resource, service delivery, sectoral collaboration, and reporting. Later, the Ministry of Health adopted the “one-plan, one-budget and one-report” approach and led efforts that resulted in improved synergy of the main global health agendas. This approach followed restructuring the budgeting and reporting scheme to one-plan, one-budget, and one-report at all levels of the health system to increase effectiveness, and played an important role in improving harmonization and alignment in the Ethiopian health sector.

### Comparison with other studies

Previous studies in low- and middle-income countries documented that weak leadership and health system have led donors to establish their own planning, implementation, monitoring, accounting, and reporting mechanisms [6–8]. This has resulted in fragmentation of service delivery and availability of medical supplies for non-commodity programs which, in turn, led the community to high out-of-pocket (OOP) spending [8, 9, 41]. The consequence of financial fragmentation was that the allocated budget was not used for the priority agendas, leading to duplications and gaps. This resulted in unmet needs in terms of geographic coverage and programs. As reported by other global health initiatives, key barriers to improving service delivery include weak drug and medical supply systems. In Sierra Leone, donors’ responses to this challenge included establishing parallel supply chains to quickly meet the needs of their specific program [9, 42]. However, unavailability of standardized and systematic indicators put tremendous burden on the available limited human resource due to duplication of effort in reporting and resulted in significant dissatisfaction [6–8].

Furthermore, pursuing equity and efficiency requires allocating resources according to health care need. However, resources were allocated based on historical precedent and political negotiation. This patchy approach to health care financing compromises equity and efficiency by hindering the effective application of the budget [43]. Thus, fragmentation is not only of concern from an equity perspective, but also in relation to health system efficiency and affordability [44].

Ethiopia’s health system has long recognized primary health care since the Alma Ata declaration and formulation of the health policy in 1993. The Ethiopian Ministry of Health took the lead in defragmenting the health sector by introducing one plan/budget/report as a useful tool for health sector planning, alignment of activities with strategic priorities and plans.

These priorities include an essential health service package to address universal health coverage proximate to the community, community-based health insurance and hospital revenue as means of health care financing, developing strategy for the health workforce, reforming the health information system and improving/assuring medical supply. A study conducted in the Ethiopian health system and health facility governance indicated that all reform efforts, including health care financing, were dependent on a well-functioning board structure [3]. Boards and governing bodies are instrumental in improving health facility performance and quality of health services [3]. Many countries in Africa are undertaking health care reforms and developing policies to improve health care systems. However, the success of health care reforms, policies and practices is dependent on the ability of the designers of health care delivery systems to replace fragmentation and waste with coordination and cost-effectiveness across disciplines [45].

Due to huge investments in health system strengthening by the Government of Ethiopia, Ethiopia was able to achieve most of millennium development goals and improved the life expectancy of its population compared to other African countries [2]. Findings from a systematic review indicated that inter-organizational relationships and linked up service delivery can improve quality and efficiency [46]. The needs of a population require collective action of organizations across the entire care continuum as they have a collective responsibility for the health and well-being of a population. This is mainly applicable to socially disadvantaged populations; those with large variations in wealth, education, culture and access to health care [46].

Studies conducted in South Africa, Tanzania and Ghana showed that community-based health insurance had been the predominant form of health insurance, but had achieved very limited coverage. These schemes only cover outpatient care at primary health-care level [44]. However, Ethiopia showed efforts to provide financial protection for the EPHS through implementing Community-based health insurance and user fee waivers at a national scale for specific care, such as TB, HIV/AIDS, leprosy, pregnancy care, family planning, fistula, and epidemics [20]. These were mechanisms designed to ensure service reach to a larger population [20].

Furthermore, the medical supply system has undergone reforms and been integrated within health system to manage all health sector supplies. This also included non-program commodities to reduce duplication and stockout and thus to strengthen efficiency of overall health systems. These findings are in line with the recommendations on systems strengthening in Sierra Leone [9].

### Strengths and limitations

Strengths of the study included the case study design which allowed triangulation between multiple data sources to map the process under study. Rich data were generated by reviewing national strategy and policy documents, and key informant interviews with the main actors in the Ethiopian health system. Findings of the key informant interviews were triangulated with strategy, policy documents and literature review. The composition of key informants was broad, to explore different insights. Due to the nature of a qualitative study, transferability is most relevant, and the findings may be useful for other countries moving towards synergy in their health system. The limitation of this study is unavailability of adequate data on the process of decision making. Further, establishing a causal link between synergistic approaches and improvement of health indicators was a challenge.

### Implications for practice, policy, and further research

#### Implication for practice

Health system fragmentation may prevent governments from ensuring universal health coverage. As stated previously, fragmentation increases inequity, inefficiency, and can lead to poor health outcomes. Inequities in health, in turn, can translate into tensions in society and threaten social cohesion and inclusion. The potential for quality service delivery rests upon leading and coordinating the various abilities of different sectors across all regions. The governance structures need increased capacity so that they can provide adequate support to the system and facility management. Competency is highly related with quality and can be obtained from different trainings and from experience. Health service needs a right balance of health workforce with different skill mix. In addition, addressing health system inequities and inefficiencies through integrated approach is likely to provide mutual benefit to both the government and community.

#### Implication for policy

Strengthening health systems, harmonizing and aligning donor funding around national health plans and strategies, following the principles of the Paris Declaration on aid effectiveness, is crucial to sustained and improved health outcomes. In addition, government’s leadership and ownership will promote health systems synergy efforts.

#### Implication for further research

The process of decision making and the continued efforts to synergy of the global health agendas need to be properly documented.

## Conclusions

In Ethiopia, fragmentation mainly manifested as inequality in accessing health services, multiple modalities of health financing, donors’ focus on funding vertical programs with poor integration to the health system, multiple actors and institutions with inadequate multi-sectoral collaboration, disparity in distribution of health workforce with high attrition rate, and variation in implementation capacity of policies and guidelines. Therefore, fostering synergies among the three global health agendas required strong stewardship and political commitment of the national government.

Ethiopia has implemented multiple approaches and instruments to overcome fragmentation. Some of the context-specific approaches used to create synergies among the three global health agendas in Ethiopia include: alignment and harmonization of donor efforts with national strategies and plans by following the principles of “One Plan, One Budget and One Report”; the Health Extension Program; a generalist approach toward health workforce development; prioritizing investment in health systems that included investment in a unified health information system and a unified pharmaceutical supply system; capital and human resource investment in creating access to primary health care; and active engagement and participation of stakeholders. Such synergy efforts may promote sustained functionality of the health system.

Finally, the negative effect of COVID-19 on essential healthcare services could challenge achieving the health-related Sustainable Development Goals. Hence, such global health issues call for a better coordinated effort between health actors to uphold approaches to synergy.

## Data Availability

The data used and/ or analyzed for the current study are available from the corresponding author, at Amare.Tadesse@lshtm.ac.uk upon reasonable request.

## Abbreviations

AIDS: Acquired Immunodeficiency Syndrome
COVID-19: Coronavirus Disease 2019
CSO: Civil Society Organizations
DHIS 2: District Health Information System 2
EHSP: Essential Package of Health Services
EPHI: Ethiopian public health institution
EPSA: Ethiopian Pharmaceuticals Supply Agency
FHAPCO: Ethiopian Federal HIV/ AIDS Prevention and Control Office
FMOH: Federal Ministry of Health
GAVI: Global Alliance for Vaccines and Immunization
GFATM: Global Fund to Fight AIDS, Tuberculosis and Malaria
GHS: Global Health Security
HEP: Health Extension Program
HP: Health Promotion
HIV: Human Immunodeficiency Virus
HIS: Health Information System
HMIS: Health Management Information System
HSDP: Health Sector Development Program
HSTP: Health Sector Transformation Plan
ICU: Intensive Care Unit
IHP: International Health Partnership
IRR: Information Revolution Roadmap
JANS: Joint Assessment for National Strategy
JCCC: Joint Core Coordinating Committee
JCF: Joint Consultative Forum
KII: Key Informant Interview
MDG: Millennium Development Goals
NCDs: Non-Communicable Diseases
NGO: Non-Governmental Organization
OOP: Out-of-pocket
PFSA: Pharmaceutical Fund and Supply Agency
PHEM: Public Health Emergency Management
PHEs: Public health emergencies
RHB: Regional Health Bureau
SDG: Sustainable Development Goals
TB: Tuberculosis
UHC: Universal Health Coverage
UNDP: United Nations Development Program
US CDC: United States Center for Disease Control
WHO: World Health Organization
